# Coronary Artery Anomalies and Anatomical Variants: Cross-Sectional Diagnostic Imaging and Clinical Background

**DOI:** 10.3390/jimaging12060273

**Published:** 2026-06-22

**Authors:** Nicolò Schicchi, Francesco Bianco, Marco Fogante, Corrado Tagliati, Luca Procaccini, Franco De Remigis, Emanuela Algeri, Giovanni Lorusso, Stefania Lamja, Giulia Argalia, Cinzia Romagnolo, Simone Steffani, Matteo Cesarotto, Luca Salice, Manuel Belgrano, Antonio Bernardini, Giuseppe Lanni, Antonio Corvino, Marcello Chiocchi, Alessandro Capestro

**Affiliations:** 1 Maternal-Child, Senological, Cardiological Radiology and Outpatient Ultrasound, Department of Radiological Sciences, University Hospital of Marche, 60126 Ancona, Italy; nicolo.schicchi@ospedaliriuniti.marche.it; 2 Department of Pediatric and Congenital Cardiology and Cardiac Surgery, University Hospital “Azienda Ospedaliero Universitaria delle Marche”, 60126 Ancona, Italy; dr.francescobianco@gmail.com (F.B.); alessandro.capestro@ospedaliriuniti.marche.it (A.C.); 3 AST Ancona, Ospedale di Comunità Maria Montessori di Chiaravalle, 60033 Chiaravalle, Italy; 4 Department of Services, UOSD Diagnostica per Immagini Teramo, Ospedale Civile Giuseppe Mazzini, 64100 Teramo, Italy; luca.procaccini93@gmail.com (L.P.); emanuela.algeri@aslteramo.it (E.A.); antonio.bernardini@aslteramo.it (A.B.); giuseppe.lanni@aslteramo.it (G.L.); 5 UOC Cardiologia, UTIC ed Emodinamica Teramo, Dipartimento Cardio-Toraco-Vascolare, Ospedale Civile Giuseppe Mazzini, 64100 Teramo, Italy; franco.deremigis@aslteramo.it; 6 Interdisciplinary Department of Medicine, Section of Radiology and Radiation Oncology, University of Bari “Aldo Moro”, 70124 Bari, Italy; lorussogiovannimd@gmail.com; 7 Diagnostica per Immagini, Fondazione Policlinico Universitario Campus Bio-Medico, 00128 Roma, Italy; stefanialamja@gmail.com; 8 Nuclear Medicine, Department of Radiological Sciences, University Hospital of Marche, 60126 Ancona, Italy; giulia.argalia@ospedaliriuniti.marche.it (G.A.); cinzia.romagnolo@ospedaliriuniti.marche.it (C.R.); 9 Dipartimento di Biomedicina e Prevenzione, University of Roma Tor Vergata, 00133 Roma, Italy; simone.soldato94@gmail.com (S.S.); marcello.chiocchi@gmail.com (M.C.); 10 Department of Radiology, Azienda Sanitaria Universitaria Giuliano Isontina Ospedale di Cattinara, 34149 Trieste, Italy; matteocesarotto3@gmail.com; 11 Department of Medical, Surgical and Health Sciences, University of Trieste, 34151 Trieste, Italy; saliceluca94@gmail.com (L.S.); mbelgrano@units.it (M.B.); 12 Medical, Movement and Wellbeing Sciences Department, University of Naples “Parthenope”, 80133 Naples, Italy; an.cor@hotmail.it

**Keywords:** coronary artery anomalies, variants, classification, ultrasound, computed tomography, magnetic resonance imaging, nuclear medicine imaging modalities, coronary angiography, intravascular ultrasound, management

## Abstract

The coronary arteries are a pair of arteries that branch off from the aorta and encircle the heart, providing oxygenated blood to the myocardium. Although coronary artery atherosclerosis remains a main cause of morbidity and mortality worldwide, coronary artery anomalies (CAAs) are increasingly recognized as a clinically relevant cause of ischemic events and can be subdivided into origin, course, or termination anomalies. The aim of this narrative review is to summarize the cross-sectional diagnostic imaging and clinical background of CAAs.

## 1. Introduction

Coronary artery anomalies (CAAs) are congenital variations in the origin, course, or termination of the coronary arteries. A variant shows more than 1% population prevalence, whereas an anomaly is less frequent. CAA are frequently asymptomatic, but arrhythmia, dyspnea, chest pain, ischemia, myocardial infarction and even sudden cardiac death have been reported. In fact, CAA can be hemodynamically significant or not [1,2].

Nowadays, coronary imaging examinations are more frequently performed and coronary variations are easily detected; these factors are related to an increasing prevalence [3]. Various imaging techniques can be performed to assess coronary artery anomalies and their functional implications: ultrasound, computed tomography, magnetic resonance imaging, single photon emission tomography, positron emission tomography, coronary angiography and intravascular ultrasound [4]. Management of these coronary anomalies and variants is strictly related to imaging findings and clinical symptoms, and sub-specialized cardiologist and cardiac surgeon referral is justified to properly treat these congenital heart diseases [1].

The aim of this narrative review is to summarize coronary artery variations’ classification, epidemiology, symptoms, diagnosis, imaging, surgical treatment and management. To support these aims, we conducted a targeted literature search. We searched PubMed/MEDLINE, Scopus, Web of Science and Google Scholar for studies published from 1 January 1980 to 30 April 2026, assessing English language studies on coronary artery anomalies using the following search strategy: (“coronary”) AND (“artery” OR “arteries”) AND (“anomaly” OR “anomalies” OR “variant” OR “variants”) AND (“diagnostic imaging” OR “imaging” OR “ultrasound” OR “computed tomography” OR “CT” OR “CCTA” OR “computed tomography angiography” OR “magnetic resonance” OR “magnetic resonance imaging” OR “MRI” OR “positron emission tomography” OR “PET” OR “single photon emission computed tomography” OR “SPECT”) and including results from the following publication types: clinical study; comparative study; controlled clinical trial; meta-analysis; multicenter study; observational study; randomized controlled trial; systematic review; case report; case series; narrative review.

## 2. Classification

### 2.1. Anomalies of Origin

**Figure 1:**
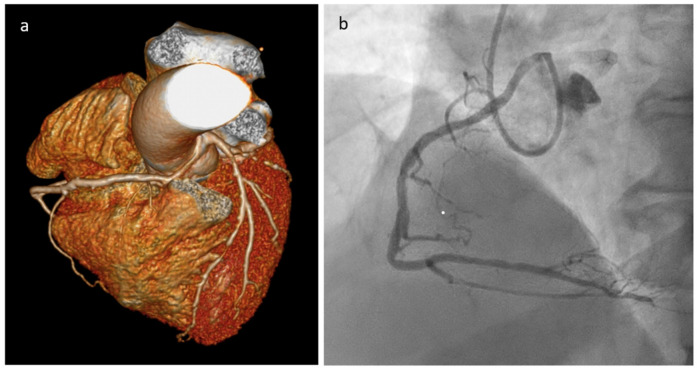
A 45-year-old male patient with a right coronary artery arising from the left coronary sinus: computed tomography volume-rendering image (**a**) and coronary angiography (**b**).

**Figure 2:**
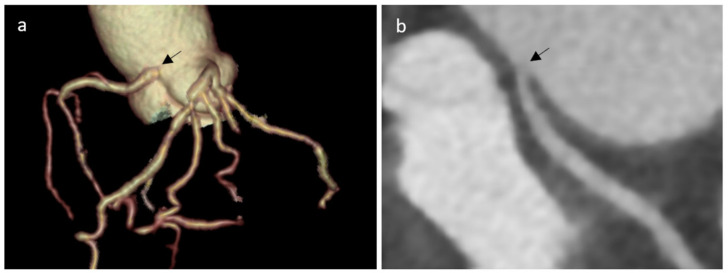
Right coronary artery from left sinus ((**a**) volume-rendering image, arrow) with slit-like ostium ((**b**) axial image, arrow) seen by computed tomography.

Anomalous aortic origin of the right coronary artery (AAORCA) typically involves the right coronary artery (RCA) arising from the left coronary sinus, with a retroaortic, interarterial or intramural course (Figure 1). The interarterial course between the aorta and pulmonary trunk may subject the vessel to dynamic compression during exercise. Ostial abnormalities—including an acute takeoff angle or slit-like orifice—further exacerbate flow limitation [5] (Figure 2).

RCA anomalous origin between the right and left coronary sinuses was described too, and in that case coronary computed tomography angiography (CCTA) demonstrated a short interarterial trajectory without other high-risk features, and subsequent cardiac magnetic resonance (CMR) identified inferior wall ischemia; therefore, the symptomatic patient was referred to the cardiothoracic surgery department for surgical unroofing [6].

**Figure 3:**
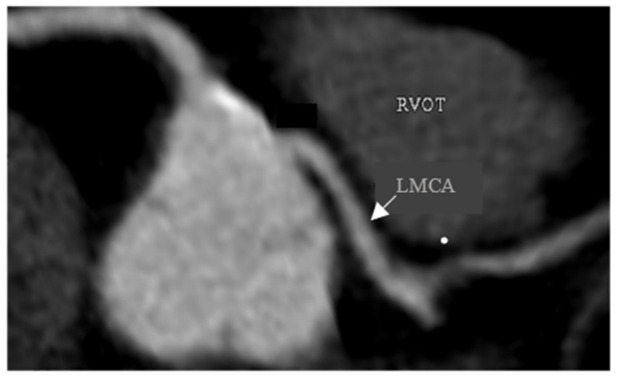
Left main coronary artery (LMCA) originating from the right coronary sinus. RVOT, right ventricle outflow tract.

Anomalous aortic origin of the left coronary artery (AAOLCA) involves the left main coronary artery (LMCA) or one of its branches originating from the right coronary sinus (Figure 3). Although rarer than AAORCA, it is more strongly associated with adverse outcomes. The LMCA may follow a retroaortic, prepulmonic, transeptal, interarterial and/or intramural course [7].

**Figure 4:**
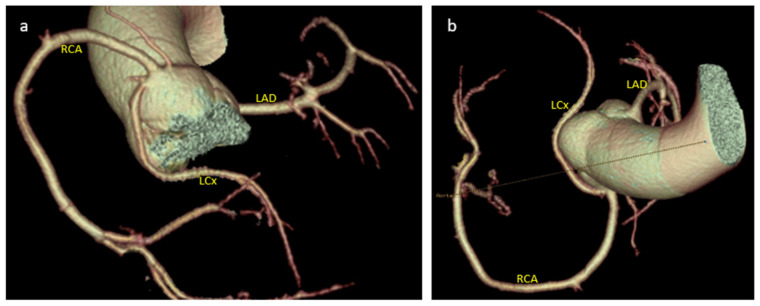
Left circumflex artery (LCx) from the right coronary sinus (**a**,**b**). RCA, right coronary artery; LAD, left descending artery.

**Figure 5:**
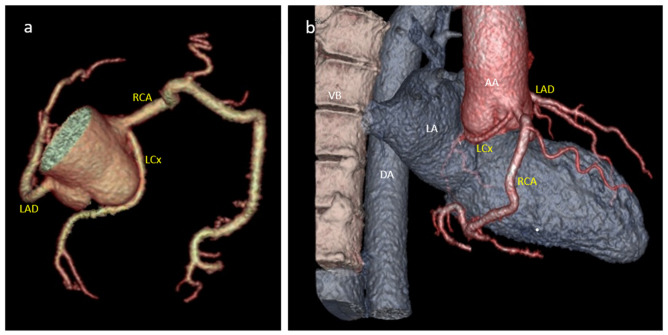
Left circumflex artery (LCx) originating from the right coronary sinus, running posterior to the aorta (retroaortic course) (**a**,**b**). RCA, right coronary artery; LAD, left anterior descending; LA, left atrium; AA, ascending aorta; DA, descending aorta; VB, vertebral body.

Anomalous origin of the left circumflex artery (LCx) may arise from the right coronary sinus or from the proximal right coronary artery, typically coursing posterior to the aorta (retroaortic course) [8] (Figure 4 and Figure 5). Type I consists in an LCx arising from a separate ostium in the right sinus, type II has a common/adjacent ostium, and in type III LCx arises from RCA as its proximal branch [9].

**Figure 6:**
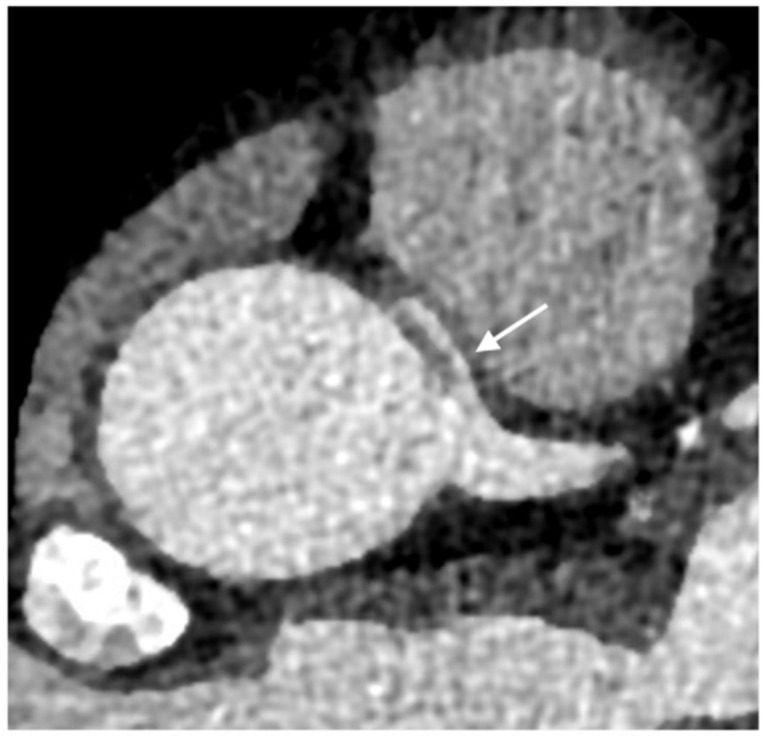
Single coronary artery from left coronary sinus with a malignant course of the right coronary artery (arrow).

**Figure 7:**
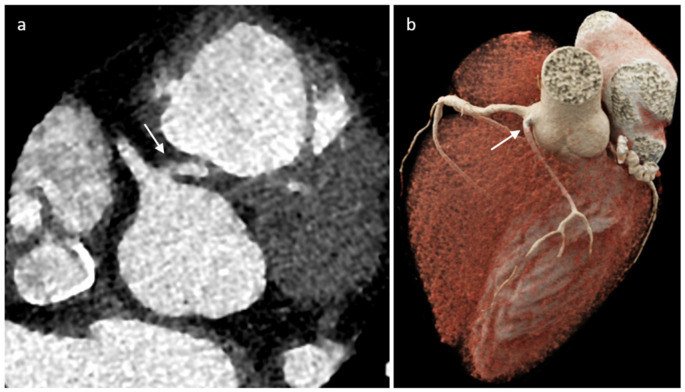
Single coronary artery from right coronary sinus with a malignant course of the left coronary artery (arrow) at axial (**a**) and volume-rendering (**b**) computed tomography images.

**Figure 8:**
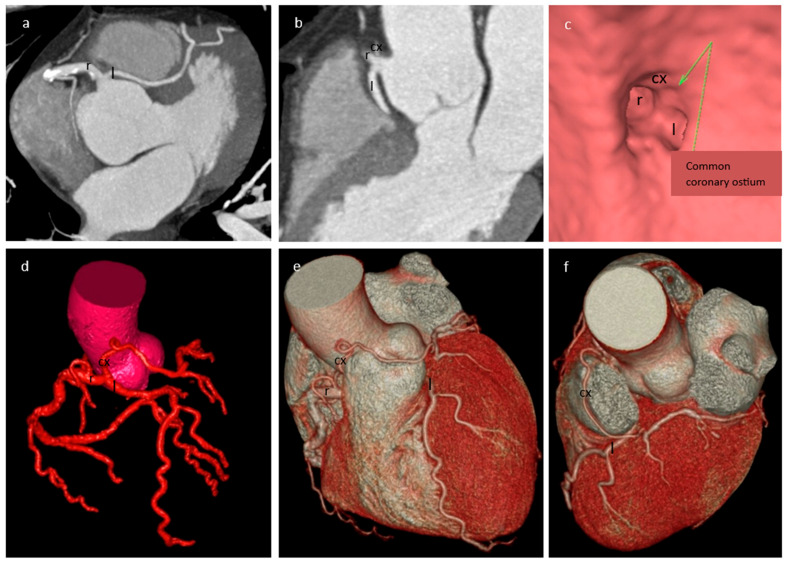
Single coronary artery from the right sinus, with a normal course of the right coronary artery (r), a malignant interarterial course of the left descending artery (l) and a pre-pulmonary course of the left circumflex artery (cx) in three-camber computed tomography images (**a**,**b**), virtual endoluminal image (**c**), and volume-rendering images (**d**–**f**).

Single coronary artery (SCA) is often associated with congenital cardiac structural deformities such as pulmonary artery atresia, tetralogy of Fallot, and patent truncus arteriosus. SCA involves the entire coronary tree originating from a solitary ostium. The vessel may divide into branches supplying all myocardial territories. The course can be benign or malignant depending on interarterial or intramural segments [10,11] (Figure 6, Figure 7 and Figure 8). Lipton classification, which was later modified by Yamanaka, is generally used and takes into account vessel ostial origin, anatomical distribution and course with respect to the pulmonary artery and the aorta; in particular, the RIIB/LIIB and RIII types respectively represent an interarterial course and right coronary artery giving rise to the left anterior descending and circumflex arteries [12,13]. A single coronary artery from an innominate artery has been described too [14].

**Figure 9:**
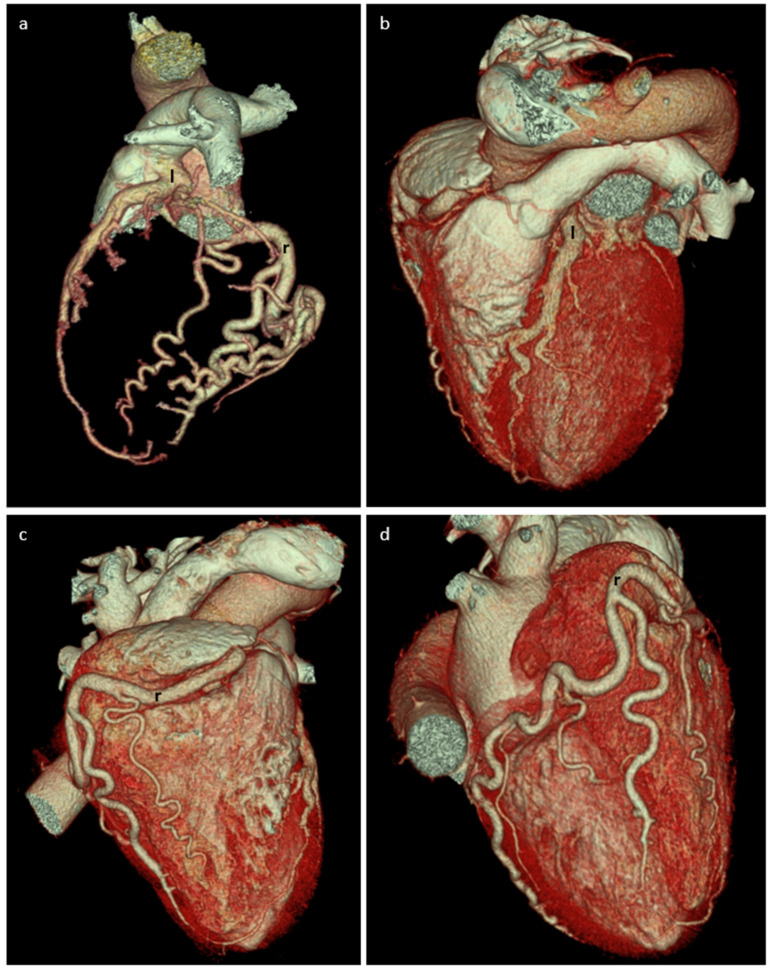
A patient with anomalous origin of the left coronary artery (l) from the pulmonary artery in computed tomography volume-rendering images (**a**,**b**), with associated dilation of the right coronary artery (r) (**c**,**d**).

Anomalous origin of the left coronary artery from the pulmonary artery (ALCAPA), also known as Bland–White–Garland syndrome, is a rare but severe anomaly in which the left coronary artery originates from the main pulmonary artery (Figure 9).

Although it is more common for the left coronary artery to arise from the pulmonary artery, an anomalous origin of the right coronary artery from the pulmonary artery (ARCAPA) can be found, with the same physiologic flow from high- to low-pressure systems taking place. Moreover, anomalous circumflex coronary artery connected to the pulmonary artery (ACXPA), anomalous left anterior descending coronary artery connected to the pulmonary artery (ALADPA), and anomalous single coronary artery connected to the pulmonary artery (ASCAPA) have been described. Furthermore, ARCAPA, ACXPA, and ASCAPA were more frequently associated with cardiac defects, particularly left heart obstructions or shunt lesions. Associated extracardiac anomalies have also been described in patients with abnormal coronary artery from the pulmonary artery [15].

**Figure 10:**
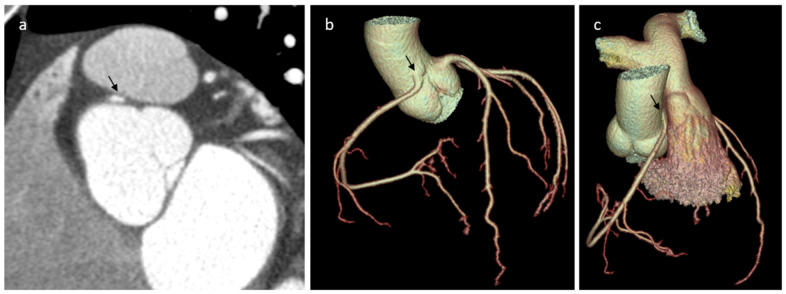
Right coronary artery high takeoff between the right and left coronary sinuses with interarterial course in axial (**a**) and volume-rendering (**b**,**c**) computed tomography images (arrows).

High takeoff refers to coronary ostia positioned more than 5–10 mm above the sinotubular junction (Figure 10). Both RCA and left coronary artery can be affected, and some associated cardiac anomalies have been described, such as double outlet right ventricle and right aortic arch [16]. High takeoff could be associated with failed attempts at cannulation in coronary angiography, and computed tomography examination could help in detecting the precise vessel origin [17].

**Figure 11:**
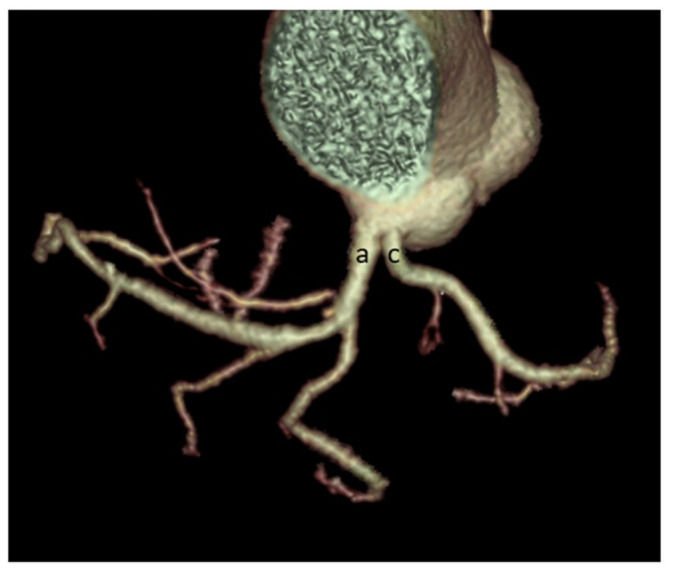
Separate origins of left anterior descending (a) and left circumflex (c) in computed tomography volume-rendering image.

Separate origins of left anterior descending (LAD) and LCx arteries mean that they originate separately from the left coronary sinus, resulting in the absence of a true left main artery [18] (Figure 11). A previously published article reported that CT demonstrated the precise, separate origin of LAD from the left coronary aortic sinus, associated with total occlusion at proximal LAD, which was not visualized at coronary angiography [19]. Moreover, few cases of a separate origin of LAD and LCx from the right sinus of Valsalva or proximal RCA have been described in the literature [20,21].

A case report showed four separate coronary artery ostia: RCA and LAD from the right sinus of Valsalva, and LCx and marginal artery from the left sinus of Valsalva [22].

Absence of LCx was rarely found, in particular with an associated super-dominant RCA; in this context, CT coronary angiography is advantageous over catheter angiography in differentiating between LCx congenital absence and its complete obstruction [23].

### 2.2. Anomalies of Course

Anomalies of course of the coronary arteries comprise a heterogeneous group of anatomical variants in which the coronary vessel, although originating from a normal or ectopic site, runs along an atypical pathway through the myocardium or between vascular and cardiac structures [24].

Regarding anomalous coronary artery from an improper sinus, the anomalous coronary artery crosses to the contralateral side of the heart in order to reach its corresponding myocardial territory. In this regard, four different courses have been described for each of these variants, and include the interarterial course, the retroaortic course, the transseptal or subpulmonic course, and the prepulmonic course [5,25]. In particular, the interarterial course the is characterized by a coronary artery which passes between the ascending aorta and the pulmonary trunk; instead, in the transseptal or subpulmonic course the coronary artery is between the aorta and pulmonary artery but with an intraseptal component [24].

**Figure 12:**
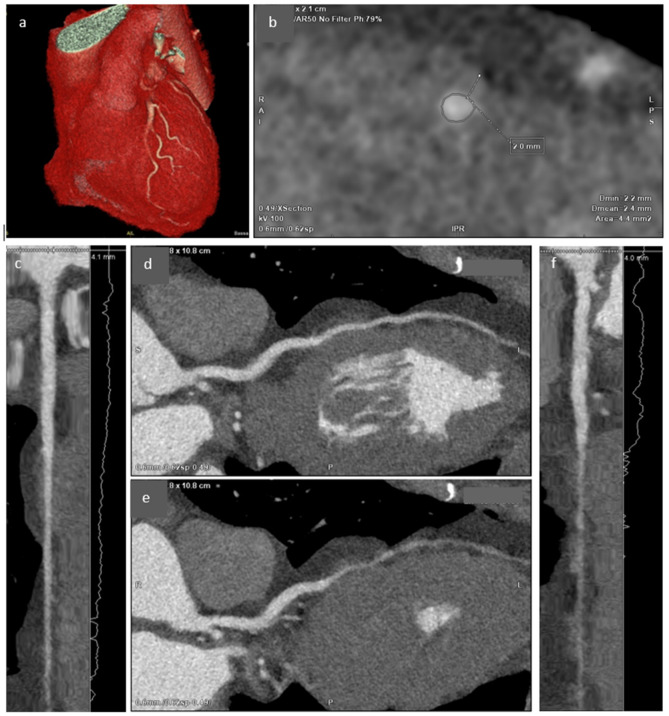
Myocardial bridging with milking effect. A myocardial bridge of the second segment of the descending artery of about 2.5 cm in length and 2 mm in depth is shown by volume-rendering image (**a**) and an axial view (**b**). Coronary curved and stretched views show the coronary diameter differences between diastolic phase (79%, (**c**,**d**)) and systolic phase (44%, (**e**,**f**)).

A distinctive course anomaly is myocardial bridging (MB), which is caused by a band of myocardial muscle overlying a segment of a coronary artery which is most commonly localized in the middle segment of the LAD. Moreover, myocardial bridging could be associated with the so-called “milking effect”, which means a significant systolic lumen reduction compared to diastolic one (Figure 12) [26].

**Figure 13:**
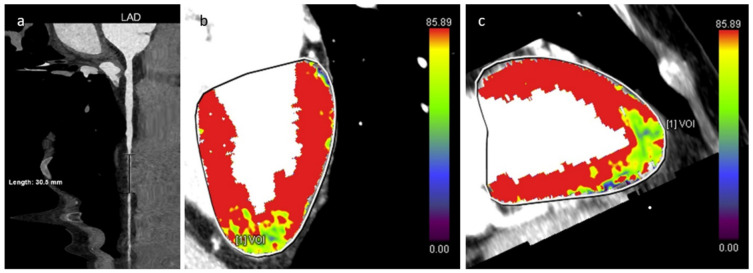
A superficial myocardial bridge which is 3 cm long (**a**) and cause apical hypoperfusion at stress dynamic computed tomography myocardial perfusion (**b**,**c**).

Coronary angiography and diastolic and systolic CCTA images can show milking effect. However, hypoperfusion can be showed by stress dynamic CT-myocardial perfusion imaging (CT-MPI), stress cardiovascular magnetic resonance imaging, single photon emission computed tomography (SPECT), positron emission tomography or invasive assessment of instantaneous wave-free ratio (iFR) [26]. MB-related hypoperfusion is more probable when high-risk anatomical features are present, in particular when the bridge is longer than 2.5 mm and its depth is at least 2 mm. A previous study reported that in patients with myocardial bridging with high-risk features, the bridge depth is the most important characteristic related to hypoperfusion at CT-MPI, with an optimal cutoff value of 3.26 mm [27]. However, superficial bridges which are long can determine hypoperfusion too [28] (Figure 13).

Ramus intermedius is a frequent coronary artery variant resulting from trifurcation of the left main coronary artery [29].

**Figure 14:**
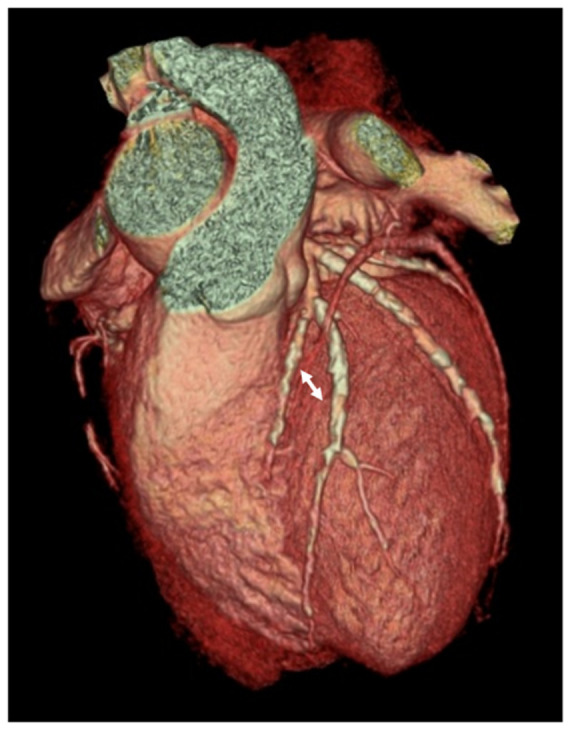
Left anterior descending artery duplication (double-headed arrow) (type I; novel classification type Ig).

**Figure 15:**
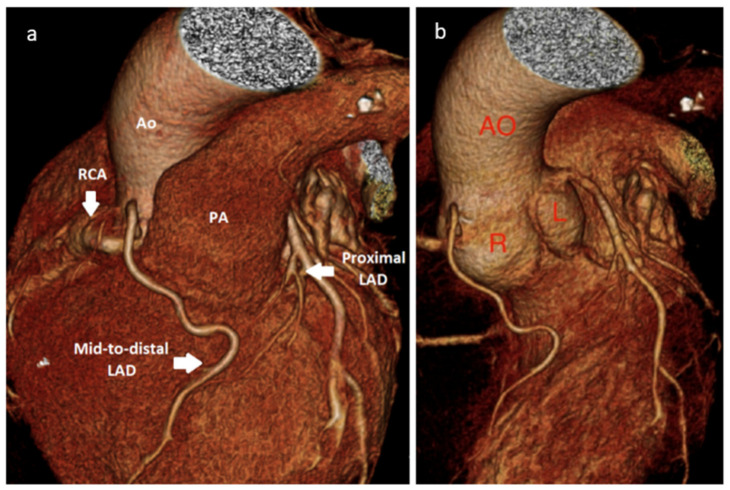
A 55-year-old male patient with an anomaly of course consistent with a dual left anterior descending artery (LAD) type IV (novel classification type IIe) in volume-rendering CT images before (**a**) and after pulmonary artery digital removal (**b**): the proximal LAD has origin from the left main trunk (**a**), while the mid-to-distal LAD (**b**) arises from the right aortic sinus (R) and reaches the interventricular groove by passing anterior to the pulmonary artery (PA), following a prepulmonic course. RCA: right coronary artery; Ao: aorta; L: left aortic sinus.

Other rare variants of coronary artery course include coronary crossing and coronary artery duplication. In coronary crossing, two branches originating from different coronary arteries intersect at the epicardial surface while being directed toward their respective myocardial perfusion territories; typically, it involves the LAD and LCx arteries which frequently arise from separate but adjacent ostia in the left aortic sinus; the second most prevalent crossing is described between LAD artery and a diagonal artery; rarely, crossing between ramus intermedius and LAD or between LCx artery and obtuse marginal branch are described [30,31]. Coronary artery duplication refers to the presence of two separate branches of the same artery, most frequently involving the LAD. Typically, a shorter branch ends in the mid segment of the sulcus interventricularis, while an accessory longer branch extends toward the apex. There are different anatomical classifications of dual LAD variants, and the first four types of dual LAD anomalies were described in 1983 by Franco et al. [32]. At the moment, the most used classification comprises about thirteen types [33]; however, a novel classification was described, which subdivided dual LAD in three groups: group I or “split” dual LAD system, where the entire left coronary artery and its major branches arise from the left coronary sinus; group II or “true” dual LAD system, where the left coronary artery originates partially from the left coronary sinus and right coronary sinus; group III or “anomalous” dual LAD system, where the entire left coronary system arises from the right coronary sinus [34] (Figure 14 and Figure 15).

Rarer variants are the intraatrial course of the right coronary artery and the intracavitary course of the left anterior descending artery [35,36].

### 2.3. Anomalies of Termination

Termination anomalies of the coronary arteries comprise a heterogeneous group of congenital abnormalities in which a coronary artery drains into an atypical cardiac or extracardiac structure [37].

The most common form is the coronary artery fistula, which can be classified according to different characteristics. One common approach categorizes them based on their site of origin, with the right coronary artery as the most frequent site of origin (50–55%), followed by the left anterior descending coronary artery (35–40%) and the left circumflex artery (5–20%) [38].

According to the classification system proposed by Sakakibara, coronary artery fistulas may instead be defined as proximal or distal based on their point of origin. When a fistula arises from the proximal third of a coronary artery, the vessel proximal to the fistula is dilated but returns to a normal caliber distal to it, conversely, when a fistula originates from the distal segment of a coronary artery, the entire vessel appears ectatic [39].

**Figure 16:**
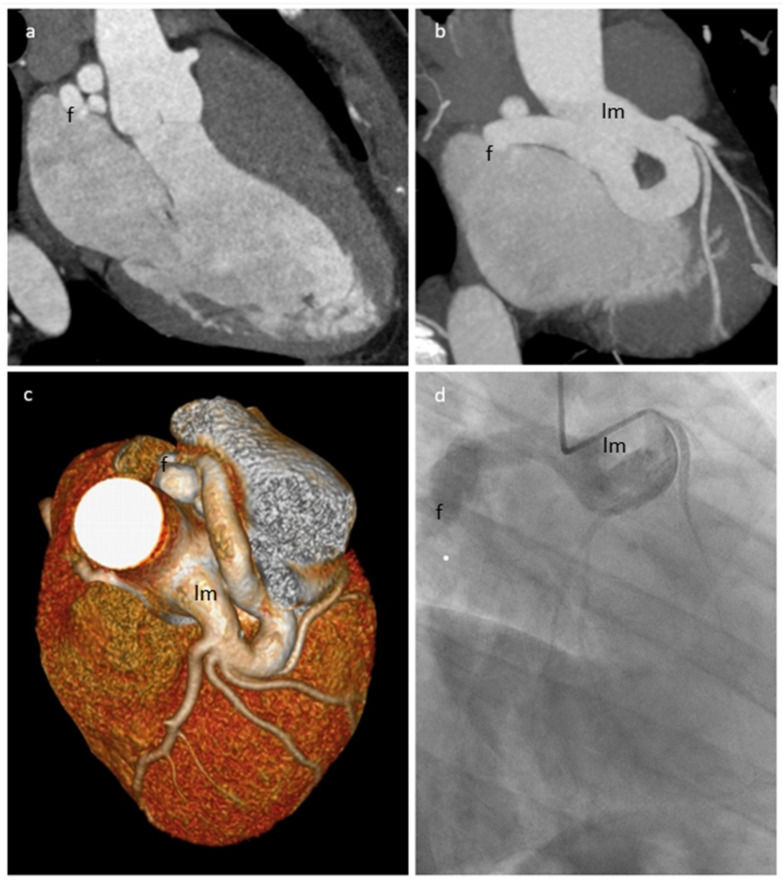
A 28 year-old male patient with a left main (lm) coronary artery to left atrium fistula (f) in computed tomography three-chamber view (**a**), curved planar reconstruction (**b**), volume-rendering (**c**), and coronary angiography (**d**).

**Figure 17:**
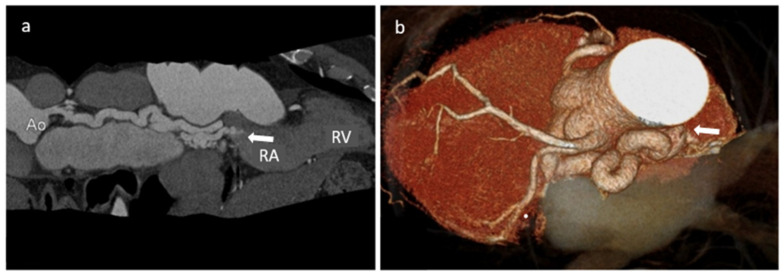
A 66-year-old male patient with a left main coronary artery to right atrium (RA) fistula (arrow) in computed tomography curved planar reconstruction (**a**), volume-rendering (**b**) images. Ao: aorta; RV: right ventricle.

**Figure 18:**
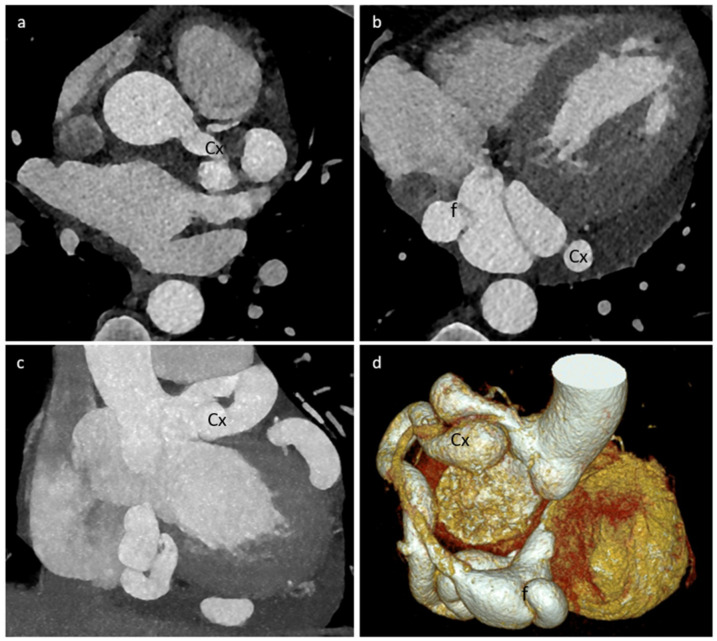
Circumflex artery (Cx) to coronary sinus fistula (f) at axial (**a**,**b**), maximum intensity projection (**c**) and volume-rendering (**d**) computed tomography images.

**Figure 19:**
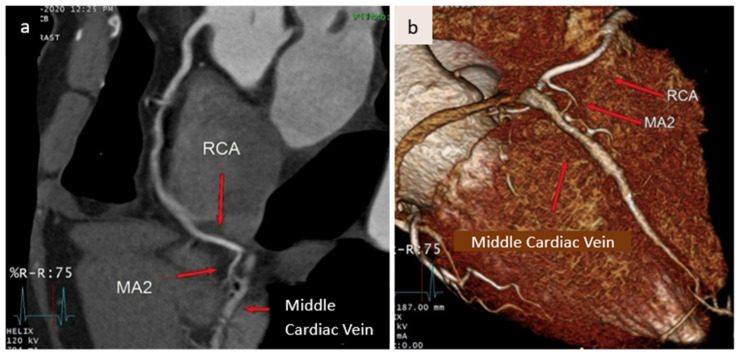
Small arteriovenous fistula between an acute marginal branch (MA2) of the right coronary artery (RCA) and the middle cardiac vein in a 58-year-old woman in computed tomography curved planar reconstruction (**a**) and volume-rendering (**b**) images.

From a clinical standpoint, one of the most clinically relevant features is the drainage site: when a fistula connects a coronary artery to a cardiac chamber, it is referred to as a coronary cameral fistula; if, instead, the fistula terminates in any vessel of the systemic or pulmonary vasculature, it is defined as a coronary arteriovenous fistula [39,40,41] (Figure 16, Figure 17, Figure 18 and Figure 19).

According to the number of fistulous tracts, coronary fistulas can be classified as single or multiple, with the former representing more than 90% of cases [37]. Coronary fistulas may commonly present in an isolated form or may coexist with other congenital heart defects [38]. Moreover, coronary fistulas can rarely be acquired after cardiac surgery, intracardiac device implantation, myocardial biopsy or trauma [40,41,42].

Extracardiac termination is another form of termination anomaly characterized by a coronary artery draining directly into a systemic artery, such as bronchial, internal mammary, pericardial, anterior mediastinal, diaphragmatic, intercostal, and esophageal arteries. Unlike true fistulas, this type of coronary termination usually does not involve an abnormal tortuous channel and does not result in arterial dilation, as frequently there is no significant pressure gradient between the coronary artery and the systemic arterial circulation [43,44,45]. A case with an extensive collateral network was reported in the literature; in particular, the anastomosis arose from the bronchial, right subclavian, and left internal thoracic arteries [46].

Intercoronary communication or coronary arcade consists of evident communications between the right and left coronary arteries through a single, wide (>1 mm), straight or gently curved vessel without proximal significant coronary stenoses, usually in the interventricular sulcus in the vicinity of the crux cordis between the anterior descending artery and the posterior descending one [43], or in the atrioventricular groove between the right coronary artery and the circumflex artery [47,48,49]; an unusual coronary artery communication between the obtuse marginal branch of the LCx and the diagonal branches of the LAD has been described too [49]; this anomaly needs to be differentiated from smaller and tortuous collateral vessels that develop between a patent and an obstructed vessel [43,50].

## 3. Epidemiology

Anomalous origins of coronary arteries from the aorta occur in about 1.5–2% of individuals [51,52,53,54]; in particular, AAORCA is reported in about 0.3–0.7% of the population, is more prevalent than left-sided anomalies of origin and is often discovered incidentally during CT-based evaluations. Anomalous origin of the LCx from the right sinus or right coronary artery is one of the most common anomalies of origin, with a prevalence of about 0.05–0.2%, and separate origins of LAD and LCx are reported in 0.4% of the population [54,55]. Instead, SCA prevalence is about 0.02–0.2% [51,54,56,57,58], ALCAPA is reported in about 0.01% of patients and ARCAPA has an estimated prevalence of 0.002% [59,60] (Table 1).

Although anomalous origins of coronary arteries from the opposite coronary sinus are relatively rare (estimated total incidence of 0.35–1.07%), the interarterial course variants can be clinically relevant as it is associated with sudden cardiac deaths [5,54,61,62].

MB prevalence varies widely depending on the diagnostic modality: it is detected in 2% of invasive angiographic studies but in 7% of CT examinations [61,63]. Therefore, MB can really be considered a coronary variant, as well as a ramus intermedius variant, the prevalence of which is about 15% [64].

Coronary artery crossing is a very rare anomaly described in 27 patients in the existing literature [30], and intracavitary right coronary artery is more frequently identified than intracavitary left anterior descending artery (0.29% vs. 0.04%) [35].

Coronary fistulas have a reported prevalence of approximately 0.4%, and the coronary arcade prevalence is about 0.002% [13,43,47,65,66].

Some previously published studies reported a higher prevalence of coronary artery anomalies in patients with other cardiac defects. Coronary anomalies are reported in about 5% of patients with tetralogy of Fallot and up to 20% of patients with truncus arteriosus [67,68]. Transposition of the great arteries, a conotruncal malformation characterized by concordance of the atrioventricular connections and discordance of the ventriculo-arterial connections, is frequently associated with coronary anomalies that should be diagnosed before arterial switch operation [67]. Coronary–cameral fistulas are reported in pulmonary atresia patients with an intact ventricular septum; moreover, left ventricle and coronary artery vascular bed connections are reported in patients with hypoplastic left heart syndrome [68].

## 4. Symptoms

Although historically considered lower risk compared with anomalous origin of the left coronary artery, AAORCA has been associated with exertional angina, syncope, ventricular arrhythmias, and rare instances of sudden cardiac death (SCD), particularly in younger or athletic individuals [69,70].

Regarding AAOLCA, given the larger myocardial territory supplied by the left coronary system, even mild dynamic obstruction may precipitate significant ischemia. AAOLCA is consistently identified as one of the coronary anomalies with the highest risk of SCD, particularly in adolescents and young athletes. Exercise-induced ischemia is common, and anomalous LMCA origin was implicated in autopsy series of sports-related cardiac deaths [61,70,71].

Regarding anomalous origin of the LCx from the right sinus or right coronary artery, its typical retroaortic course is generally considered benign; in fact, most individuals remain asymptomatic, and ischemia is rare unless concomitant atherosclerosis exists [72].

SCA clinical impact depends on the course: interarterial trajectories (the RIIB/LIIB and RIII types) confer increased risk of ischemia, arrhythmias, and, in some cases, SCD. Benign variants may remain asymptomatic throughout life [10,12,13,73].

In ALCAPA, postnatal decline in pulmonary artery oxygen saturation leads to chronic myocardial ischemia, left ventricular dysfunction, and the development of extensive collateral networks from the right coronary artery. Infants typically present with heart failure, mitral regurgitation, or failure to thrive. In rare adult survivors, cardiomegaly and chronic ischemia with reduced left ventricular ejection fraction may manifest as angina, arrhythmias, or sudden death [74].

High takeoff alone is typically considered benign, although it may complicate catheter-based coronary angiography or perioperative aortic procedures. However, considering only the coronary arteries arising at least 1 cm in adults or 20% of the depth of the sinus in children above the sinotubular junction, a higher rate of SCD was reported; therefore, these parameters are considered of greater clinical relevance [75,76,77]. Sometimes, ostial stenosis or acute angulation may contribute to ischemia [78,79].

Separate origins of LAD and LCx are generally benign and often discovered incidentally. There is typically no association with myocardial ischemia unless additional anomalies or atherosclerotic disease are present [21,22,80,81].

While most of the anomalies of course are not hemodynamically relevant, certain configurations may be associated with myocardial ischemia, ventricular arrhythmias, or, more rarely, sudden cardiac death. In fact, based on pathophysiological criteria, the interarterial course represents a hemodynamically significant anomaly, as the coronary artery passes between two high-pressure vascular structures, thereby becoming particularly susceptible to extrinsic dynamic compression, especially during physical exercise, which can impair coronary flow. The risk is further increased by the presence of aggravating anatomical factors, such as an intramural course within the aortic wall, a slit-like coronary ostium, acute-angle takeoff or an orifice >1 cm above the sinotubular junction [82].

Other variants, such as the retroaortic and prepulmonic courses, are considered morphologic anomalies without a significant impact on coronary flow and are therefore classified as non-hemodynamically relevant [43]. Trans-septal/subpulmonic course is generally considered benign; however, fatal cases, arrhythmia, and myocardial ischemia have been reported in the literature [83,84,85].

During systole, the intramyocardial segment of a myocardial bridging may undergo dynamic compression, leading to a transient reduction in coronary blood flow. Although generally considered a benign and asymptomatic finding, myocardial bridging may result in myocardial ischemia, ventricular arrhythmias, or, rarely, sudden death, particularly when its depth is more than 5 mm (very deep bridges) and its length is more than 25 mm [84]. Moreover, atherosclerotic plaques are more frequent in the segment just proximal to the myocardial bridge than the myocardial bridge itself, which is usually protected against the development of atherosclerosis due to the shear stress caused by myocardial contraction on the arterial wall, as well as the different intimal histological structure with less cell proliferation and collagen deposition [28,63,86,87].

The ramus intermedius variant is not related with specific symptoms per se; however, a higher plaque burden in the LMCA is reported in the literature [88].

Coronary crossing and coronary artery duplication are morphologic anomalies without clinical significance but can be relevant in diagnostic and preoperative contexts [28].

Intracavitary course is relatively benign, with absent symptoms and normal prognosis [35,89].

Many small fistulas are asymptomatic, and they account for about 75% of cases [37]; however, when altered blood flow dynamics are consistent, fatigue, dyspnea, typical or atypical chest pain, arrhythmia, pre-syncope or syncope can be reported by patients, sometimes associated with a heart murmur upon physical examination [90].

Intercoronary communication is generally not related with ischemia; however, rarely, coronary steal with unidirectional flow can cause ischemic symptoms [91,92].

## 5. Diagnosis

CAA presence can be suspected in young patients with ischemia-like symptoms. However, CAA diagnosis is most incidental. Ultrasound is usually the first imaging modality due to its availability and is frequently performed in pediatric patients too, allowing us to suspect it; computed tomography is frequently considered the noninvasive imaging reference, due to its detailed anatomical assessment and quick acquisition; magnetic resonance imaging is a complementary imaging technique but is invaluable for noninvasive ischemia and infarction detection; nuclear medicine can sometimes be used to obtain noninvasive functional data; invasive coronary angiography is a valuable presurgical tool, particularly if intravascular ultrasound is performed for ostium and potential intramural course evaluation [93,94].

Obviously, a clear differential diagnosis should be performed. The most important one is coronary artery atherosclerosis, which can be the cause of symptoms in patients with coronary anomalies too. To obtain these data noninvasively, CCTA is the imaging reference due to its inherent capacity to give a detailed anatomical vascular and plaque depiction. Obviously, sometimes invasive imaging is necessary to clearly evaluate functional implications of potential significant coronary plaques. Moreover, it should be noted that CAAs are frequently reported as incidental findings during workup for ischemic heart disease [93,94,95].

In fact, AAORCA is often discovered incidentally during CT-based evaluations, but sudden cardiac death is reported in the literature; moreover, anomalous coronary artery atherosclerosis could obviously cause symptoms and myocardial infarction, with subsequent anomaly detection [96,97,98]. LMCA anomaly diagnosis could sometimes be obtained only during autopsy as it was implicated in autopsy series, particularly in sports-related cardiac deaths [99,100,101,102]. Early diagnosis of an anomalous coronary artery connected to the pulmonary artery can substantially improve outcomes and heart murmur, dyspnea or palpitations could be signs of this anomaly; a widened and tortuous coronary artery can be detected at ultrasound, and subsequent imaging techniques can confirm the diagnosis to decide proper management [103,104,105].

High takeoff coronary arteries and separate origins of LAD and LCx are frequently detected incidentally during CCTA or coronary angiography examinations [106,107,108,109].

Atypical chest pain on exertion or syncope can be symptoms of an interarterial course and, given its high clinical risk, an early diagnosis with accurate anatomical assessment is crucial, not only to define the coronary origin and course but also for appropriate risk stratification and selection of the most suitable therapeutic approach [110,111,112]. Retroaortic, transseptal/subpulmonic, and prepulmonic courses are usually identified incidentally during coronary imaging examinations [37,113].

CCTA can easily detect an MB, but the extent of coronary flow impairment depends on several factors, including the length and depth of the tunneled segment. Functionally, a three-grade classification has been proposed based on the percentage of systolic luminal narrowing: no systolic compression, systolic compression < 50%, and systolic compression ≥ 50%) [114]. However, drugs administered to patients prior to CCTA have the potential to mask the clinical importance of MBs. Therefore, CCTA stress perfusion can be performed to overcome this issue [27]. Moreover, a transluminal attenuation gradient and CCTA-derived fractional flow reserve could help to define MB functional implications; the former evaluates the luminal attenuation gradient (i.e., the change in Hounsfield units per 10 mm length of the coronary artery) and the latter involves a computational fluid dynamics simulation analyzing adenosine-induced hyperemia in conjunction with CCTA imaging, in particular obtaining the difference in CCTA–fractional flow reserve (FFR) values between the proximal and distal MB ends [114,115]. Obviously, stress cardiovascular magnetic resonance imaging, SPECT and positron emission tomography can assess inducible ischemia and coronary microvascular dysfunction, like iFR during coronary angiography [116].

The ramus intermedius variant, coronary crossing and coronary artery duplication are generally incidentally diagnosed upon imaging examinations [64,88]. However, ramus intermedius is associated with higher LMCA and proximal LAD atherosclerotic plaque prevalence and stenosis degree, at least partially related to hemodynamics and wall shear stress changes [64,88,117].

An intracavitary course is usually accidentally discovered in a pre-operative CT angiography or during surgery [35], and preprocedural detection of an intraatrial or intraventricular course is paramount for proper planning to avoid arterial injury during radiofrequency ablation too [118].

Fistulas and intercoronary communications are usually incidentally detected during a computed tomography examination or coronary angiography [50,90]. However, rarely, fistulas can alter blood flow dynamics causing murmur and symptoms such as dyspnea, heart failure, angina, and arrhythmias, and dilated and tortuous vessels can be seen upon echocardiography [90,119].

## 6. Imaging

Diagnostic imaging can provide morphological and functional information; the former can be obtained with ultrasound, CCTA and MRI; the latter can be acquired with CCTA-FFR, CT and MR perfusion imaging, and nuclear medicine. Both morphological and functional information can be obtained by invasive coronary angiography, with better morphological data about ostial narrowing and intramural segment obtained by intravascular ultrasound [93,94].

### 6.1. Ultrasound

Historically, echocardiographic evaluation of the coronary arteries has always been considered extremely operator-dependent and inaccurate when acoustic windows are suboptimal; however, it may be appropriate as the first imaging technique when a coronary artery anomaly is suspected [120,121].

Four main acoustic windows can be performed to evaluate the coronary arteries. The parasternal short axis (PSAX) is the traditional approach for coronary origin and proximal course assessment. In fact, a hallmark of anomalous aortic origin of a coronary artery is considered the lack of proper visualization of its origin from the aortic root in PSAX [120]. However, coronary arteries can be assessed in the parasternal long axis (PLAX) and apical fourth and fifth chamber views too, in the latter focusing on the aortic root [122,123,124].

In PLAX, the presence of the so-called ring sign should be checked; if present, it should be interpreted as atypical. This sign is of interest when an anomalous origin from the opposite coronary sinus and an intramural or intraarterial course is suspected. Indeed, a coronary artery originating from the contralateral aortic sinus crosses the mid-anterior line, a feature that may be visible in the PLAX view [120].

Under normal conditions, by tilting the transducer posteriorly from the PLAX and focusing on the right ventricular outflow tract, it is possible to visualize the left main artery and the left anterior descending artery. An anomalous origin of the anterior descending artery from the right sinus of Valsalva should be suspected on this projection if both the anterior descending artery and the right anterior coronary artery are visualized simultaneously. Furthermore, the failure to visualize the anterior descending artery on this acoustic window may suggest its agenesis or hypoplasia [120].

**Figure 20:**
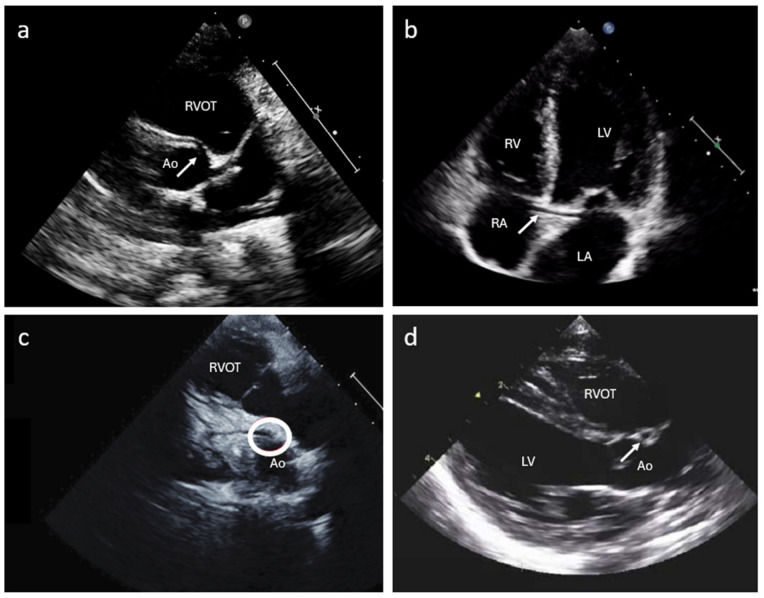
Ultrasound examination in coronary anomalies: (**a**) Right coronary artery from the left sinus of Valsalva in parasternal short-axis view (arrow). (**b**) Circumflex coronary artery from the right sinus of Valsalva with a retroaortic course in four-chamber view (arrow). (**c**) Single coronary artery from a modified parasternal short-axis view (circle). (**d**) The ring sign indicates a right coronary artery from the opposite sinus of Valsalva with a presumed intramural course on parasternal long-axis view (arrow). RVOT, right ventricular outflow tract; LV, left ventricle; RV, right ventricle; Ao, aorta, LA, left atrium; RA, right atrium.

On the apical four-chamber view, if a coronary artery is visualized with a dual-track image, the so-called retro-aortic sign (RAC), an anomalous origin of the circumflex artery from the right coronary sinus with a retro-aortic course should be suspected [122] (Figure 20).

Anomalous origins from the pulmonary artery do not present a predictable pattern; however, the absence or failure to visualize a coronary artery (PSAX, PLAX, fourth and fifth chambers views), the visualization of a dilated coronary artery in the atrioventricular grooves (PLAX, apical fourth and fifth chambers), or a coronary artery apparently originating from the pulmonary artery should raise suspicion [123].

To correctly visualize normal coronary arteries and anomalous origins, traditional two-dimensional echocardiography can and should be used, optimizing near- and far-field grayscale imaging, as well as color Doppler imaging. Since coronary arteries typically exhibit low-velocity flow, the Nyquist limit should be reduced to 20–40 cm/s, as recommended [120,123,125]. Finally, the probe with the least penetrating power but greater proximal definition (higher MHz) should be chosen in pediatric patients, and the classic phased-array probe in adults. Where transthoracic images are suboptimal to confirm the diagnosis, sometimes transesophageal echocardiography can be performed; the latter can also help in identifying coronary artery fistulas [124,126].

### 6.2. Computed Tomography

CCTA has become the primary noninvasive modality for diagnosing CAAs because it combines high spatial resolution, fast volumetric coverage and multiplanar/3D reconstructions that clearly show the origin, course, and relationships of anomalous vessels to surrounding cardiac structures—features that are often difficult or ambiguous upon invasive angiography alone [127]. The scan range should extend from the aortic arch to the diaphragm to capture high takeoff and pulmonary–aortic relationships [128]. Historically, limitations for coronary imaging were motion blur (temporal resolution), limited spatial resolution for small-caliber vessels and relatively high radiation/contrast doses; modern hardware and software advances directly address these issues and therefore materially improve CCTA’s ability to detect clinically important features of CAA such as intramural course, interarterial trajectories, ostial morphology and precise relation to the aortic root and pulmonary artery [129]. Moreover, an intracavitary course can be diagnosed if a segment is seen completely surrounded by intracavitary contrast in all phases of the cardiac cycle [35]. Dual-source CT (DSCT) was one of the first major hardware steps forward: by using two X-ray sources and detectors at orthogonal positions, DSCT effectively doubles temporal sampling and reduces the temporal resolution requirement for capturing the rapidly moving coronaries, so that high-quality coronary datasets can be obtained at higher heart rates or with fewer/less aggressive rate-control medications, an important practical advantage when scanning symptomatic or unstable patients or children with higher native heart rates [130,131]. This improved temporal resolution reduces motion artifacts that could obscure an anomalous vessel’s proximal ostium or short intramural segments and thus increases diagnostic confidence when defining potentially malignant courses [132,133]. Equally important are reconstruction advances: iterative reconstruction (IR) algorithms (statistical IR, model-based IR, or vendor-specific hybrids) have allowed substantial radiation dose reductions while preserving or improving vessel contrast-to-noise and edge definition compared with filtered back projection. In the context of CAA, the improved noise performance of IR makes it easier to delineate small anomalous branches, to assess vessel tapering and to characterize the vessel wall near complex ostia or calcified origins—all while enabling routine low-dose protocols that are preferable in younger patients who often present with congenital anomalies [134,135]. Beyond dose and noise, IR and advanced post-processing also reduce blooming from calcifications and stents, improving lumen assessment in partially calcified anomalous vessels and reducing false-positive stenosis estimates that could complicate management decisions [136]. The most recent and potentially disruptive advance is photon-counting detector CT (PCCT): unlike conventional energy-integrating detectors, photon-counting detectors register individual X-ray photons and their energy, yielding intrinsically higher spatial resolution, reduced electronic noise, improved contrast-to-noise ratio, and multi-energy (spectral) information without the penalties of dual-energy switching or noise amplification. For coronary imaging, this translates into crisper depiction of small-caliber vessels and intramural segments, better delineation of ostial geometry, and improved plaque characterization (for example improved discrimination of lipid-rich versus fibrous components), which can be decisive when assessing whether an anomalous vessel has atherosclerotic involvement in its proximal segment or an intramural narrowing. Early human and ex-vivo studies show that PCCT reduces overestimation of stenosis and improves visualization of the in-stent lumen and vessel wall microstructure, advantages that are directly applicable to identifying high-risk features in CAA (e.g., slit-like ostium, acute takeoff, intramural narrowing) [137,138]. Practically, combining these elements yields a modern CCTA strategy for CAA: use wide-detector or DSCT platforms (or PCCT where available) with prospectively gated high-pitch or targeted ECG gating to minimize dose and motion, pair acquisitions with low-kV protocols and contrast timing optimized to the coronary circulation, and reconstruct with the latest IR or model-based algorithms (and, if available, photon-counting reconstructions and virtual monoenergetic images) to maximize lumen contrast and spatial detail while keeping radiation as low as reasonably achievable—particularly critical given that many patients with congenital anomalies are younger [134,139,140].

### 6.3. Magnetic Resonance Imaging

**Figure 21:**
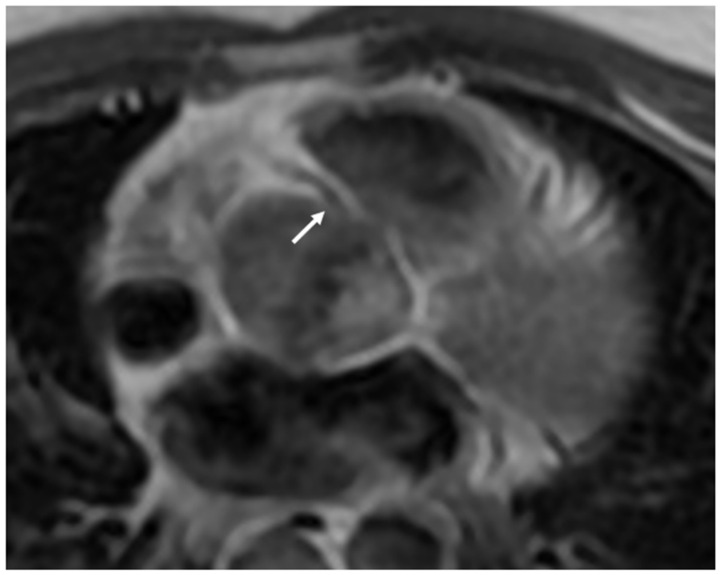
Right coronary artery origin from left sinus with intraarterial course (arrow) in magnetic resonance imaging.

CMR represents a valuable diagnostic tool in the evaluation of coronary artery anomalies in both pediatric and adult populations, particularly for the identification of anomalous coronary origins and when it is desirable to avoid ionizing radiation or iodinated contrast media [121]. Despite its high contrast resolution and multiplanar imaging capabilities, CMR has intrinsic limitations in the accurate assessment of the coronary arterial course and distal segments, mainly related to spatial resolution, cardiac and respiratory motion, and the complex anatomy of the coronary tree. Therefore, its role should be usually considered complementary to other imaging modalities in the comprehensive characterization of coronary artery anomalies [5,141]. However, an unexpected coronary artery anomaly can be found during MRI examination in elite athletes too [141,142,143] (Figure 21).

At 1.5 T, coronary imaging typically relies on balanced steady-state free precession (bSSFP) sequences, which provide excellent blood–myocardium contrast and uniform vessel visualization [144]. At 3 T, however, conventional gradient-echo techniques combined with magnetization preparation (e.g., T2-preparation or inversion recovery) are often preferred to suppress background signal and mitigate bSSFP banding artifacts [145]. Most contemporary protocols incorporate fat suppression—either through spectral saturation or more advanced off-resonance pulses—to nullify the epicardial fat signal. For instance, lipid-insensitive binomial pulses (LIBRE and the newer LIBOR) have demonstrated effective, homogeneous whole-heart fat suppression at 3 T, while reducing both SAR and acquisition time. More sophisticated approaches, such as dual-phase imaging (simultaneous systolic and diastolic acquisition), have been explored to ensure that coronary anomalies are visualized in their optimal cardiac phase. Recent technical innovations have further advanced coronary CMR. Self-navigation and image-based navigator (iNAV) methods now enable efficient, motion-corrected, free-breathing acquisitions. Henningsson et al. reported that iNAV-enhanced CMR angiography yielded diagnostic-quality images in 98% of proximal coronary segments, achieving ~86% sensitivity and 83% specificity for detecting coronary stenosis [146]. The shift toward higher field strengths (3 T and even investigational 7 T) offers potential gains in SNR and submillimeter spatial resolution, though 7 T remains primarily a research tool [145]. Furthermore, artificial intelligence and deep learning–based reconstructions are beginning to influence coronary CMR workflows. Montalt-Tordera et al. demonstrated that a residual U-Net-based reconstruction of accelerated, noncontrast CMR angiography achieved 88% sensitivity and 96% specificity for detecting vascular abnormalities, allowing an ~80% reduction in contrast dose [147].

Recent studies have confirmed the high diagnostic accuracy of CMR for congenital CAAs. In a prospective study of 65 pediatric patients with suspected CAAs, 3D whole-heart CMR using either diaphragmatic navigation (dNAV) or self-navigation (sNAV) achieved 96.8% sensitivity and 100% specificity compared with CCTA or invasive angiography [148]. Similarly, in a series of 78 adults (17 with CAAs), CMR correctly identified or excluded all anomalies, yielding 100% diagnostic accuracy [149]. Earlier studies in smaller pediatric cohorts reported somewhat lower performance: for example, in 21 children imaged with 3D radial sNAV CMR at 1.5 T, sensitivity and specificity were 71% and 92% for high-risk anomalies, and sensitivity was 92% for overall CAA detection [150].

In fact, spatial resolution limitations may restrict the evaluation of distal coronary segments, making coronary CTA the preferred modality when detailed delineation of the distal vessel course is required. However, flow-sensitive CMR sequences can also quantify shunt magnitude in the setting of coronary–cameral fistulas [151,152].

CMR remains valuable for comprehensive myocardial assessment—including the evaluation of viability and ischemia—in the preoperative evaluation of CAAs, particularly when combined with stress perfusion imaging [121,153,154]. In fact, stress CMR is feasible in these patients, and it could be also performed during in vivo exercise using supine cycle ergometry where CMR-compatible exercise equipment is available, avoiding potential side effects of pharmacologic stress testing [155].

### 6.4. Nuclear Medicine Imaging Modalities

Nuclear cardiology techniques, particularly single-photon emission computed tomography (SPECT) and positron emission tomography (PET), provide information on their functional implications such as regional myocardial perfusion, ischemia, viability, and physiological flow reserve. When performing these tests with radioactive drugs, especially in children, it is essential to pay special attention to radiation (as low as reasonably achievable), weight-based radiotracer dosing, short scan times and advanced reconstruction algorithms to maintain diagnostic quality with minimal exposure [156,157].

**Figure 22:**
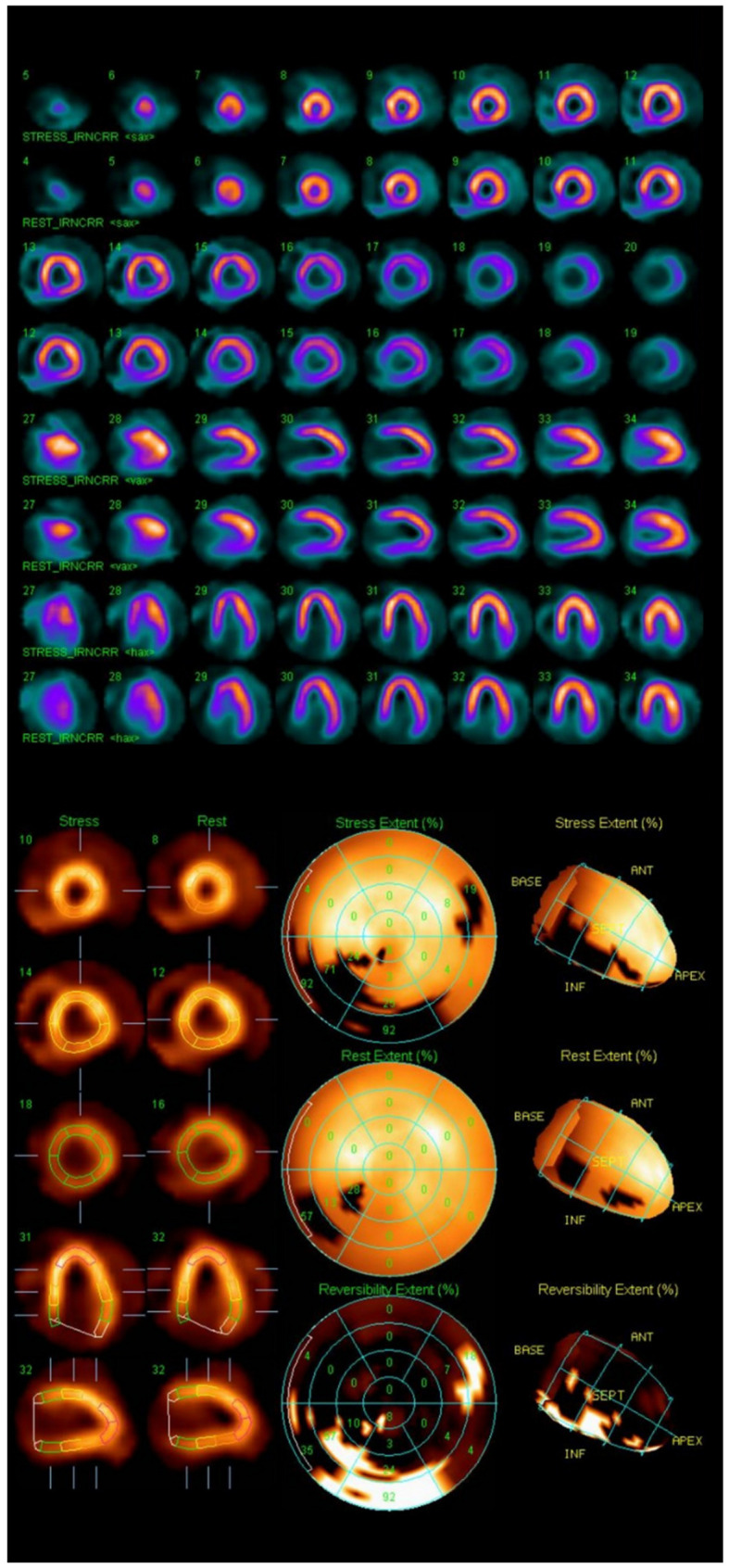
A 65-year-old male patient with anomalous origin of the right coronary artery from the left/posterior coronary sinus of Valsalva associated with an anomalous/malignant intraarterial proximal coronary course of the right coronary artery (between the pulmonary artery anteriorly and the aorta posteriorly; probable intramural nature of the paraostial coronary segment). Myocardial perfusion SPECT with technetium-99m tetrofosmin performed after pharmacologic stress with regadenoson shows a moderate myocardial hypoperfusion in the infero-septal area, almost completely reversible in the rest images, consistent with stress-induced ischemia in the right coronary artery territory. Left ventricular dilation with mild reduction in contractile function.

**Figure 23:**
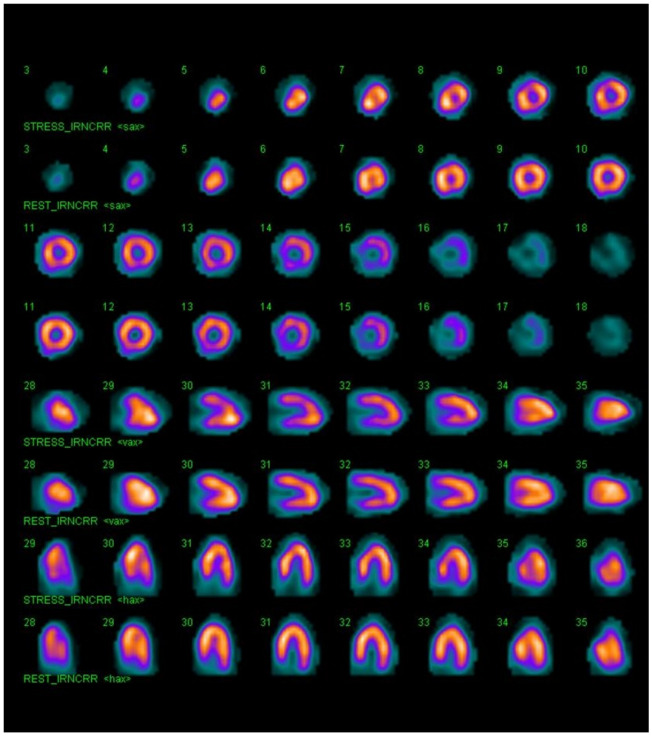
A 59-year-old male patient with complete muscle bridge on the third segment of the anterior descending artery (IVA 3). Myocardial perfusion SPECT with technetium-99m tetrofosmin performed after pharmacologic stress with regadenoson and at rest demonstrates homogeneous tracer uptake throughout all myocardial segments, with no evidence of reversible or fixed perfusion defects. Left ventricular size and function are within normal limits. Ejection fraction is preserved.

SPECT myocardial perfusion imaging (MPI) using 99mTc-based tracers (99mTc-sestamibi and 99mTc-tetrofosmin), and less commonly 201Tl, remains a widely available method for the assessment of regional perfusion and ventricular function. Standard protocols employ stress–rest acquisitions with exercise or pharmacologic stress (adenosine, dipyridamole, regadenoson, or dobutamine where appropriate) and gated acquisitions that allow simultaneous evaluation of the left ventricular ejection fraction and wall motion [68]. In CAAs, SPECT MPI is useful for detecting reversible regional perfusion defects (related to the territory supplied by an anomalous vessel) consistent with stress-induced ischemia, for identifying non-reversible defects representing previous infarction, and for quantifying the extent of ischemic burden for surgical decision-making (Figure 22 and Figure 23). Hybrid attenuation-corrected SPECT/CT improves diagnostic accuracy by reducing soft-tissue attenuation artifacts (e.g., diaphragmatic or breast attenuation). Advances in detector technology (e.g., cadmium–zinc–telluride) and iterative reconstruction methods have improved SPECT spatial resolution and count sensitivity, allowing us to reduce the administered activity [156] or to decrease the duration of image acquisition [158,159,160]. SPECT MPI technology allows a semi-quantitative estimation of myocardial perfusion, but until now has not permitted absolute myocardial blood flow (MBF, mL/min/g) measurements. Pitfalls include balanced ischemia and attenuation/motion artifacts. Despite this, being a low-cost and widely available technology, it is still the most used in clinical practice.

PET MPI offers higher spatial resolution than SPECT, demonstrates superior diagnostic performance in the detection of multivascular or microvascular dysfunction, and, importantly, enables quantification of perfusion, allowing the absolute calculation of MBF at rest and during pharmacological hyperemia, as well as quantification of myocardial flow reserve (MFR) [161,162]. In particular PET is the gold standard for a noninvasive quantitative assessment of MBF [163]. Common clinical perfusion tracers include [82Rb]Cloruro (generator-produced), [13N]Ammonia (cyclotron-produced), and [15O]Water (research/tertiary centers). Unfortunately, these radiotracers have short half-lives, are extremely expensive, and are not widely available. Emerging 18F-labeled tracers (e.g., [18F]Flurpiridaz) promise wider distribution and improved counting statistics in the near future [164,165] (Table 2). Quantitative PET MPI is expected to become central to the functional assessment of CAA, particularly for lesions with equivocal relative perfusion or suspected microvascular involvement. Additionally, [18F]FDG-PET imaging is used to assess myocardial metabolism and viability, guiding expectations for functional recovery after surgical correction [165,166,167,168]. Hybrid PET/CT and PET/MRI facilitate precise co-localization of anatomic coronary anomalies with perfusion and metabolic abnormalities. The most comprehensive noninvasive assessment could be provided by hybrid PET/MRI systems that integrate flow quantification with scar imaging and tissue characterization [159] (Table 3).

### 6.5. Coronary Angiography and Intravascular Ultrasound

Coronary angiography is reserved for interventional procedures or when noninvasive assessment is inconclusive, such as in patients with conflicting data between imaging and symptoms, and functional invasive tools are needed [94,169].

A milking effect can be easily detected in coronary angiography in an intramural myocardial bridge or, rarely, in an intracavitary course when an abnormally elevated right ventricular systolic pressure may exceed the coronary artery pressure [35].

Moreover, invasive functional evaluation can be performed to grant sports activity in patient without high-risk anatomic features. Intravascular ultrasound (IVUS) can calculate effective coronary lumen area and its changes during the cardiac cycle and pharmacologic stress tests using dobutamine in pediatric patients too [170].

FFR represents the ratio between mean coronary artery pressure distal to a stenosis and mean aortic pressure measured simultaneously in the aortic root, and it can be evaluated used during stress tests too; current evidence on atheromatic plaque settings indicates 0.80 as the cutoff value for significant stenotic lesions [171].

**Figure 24:**
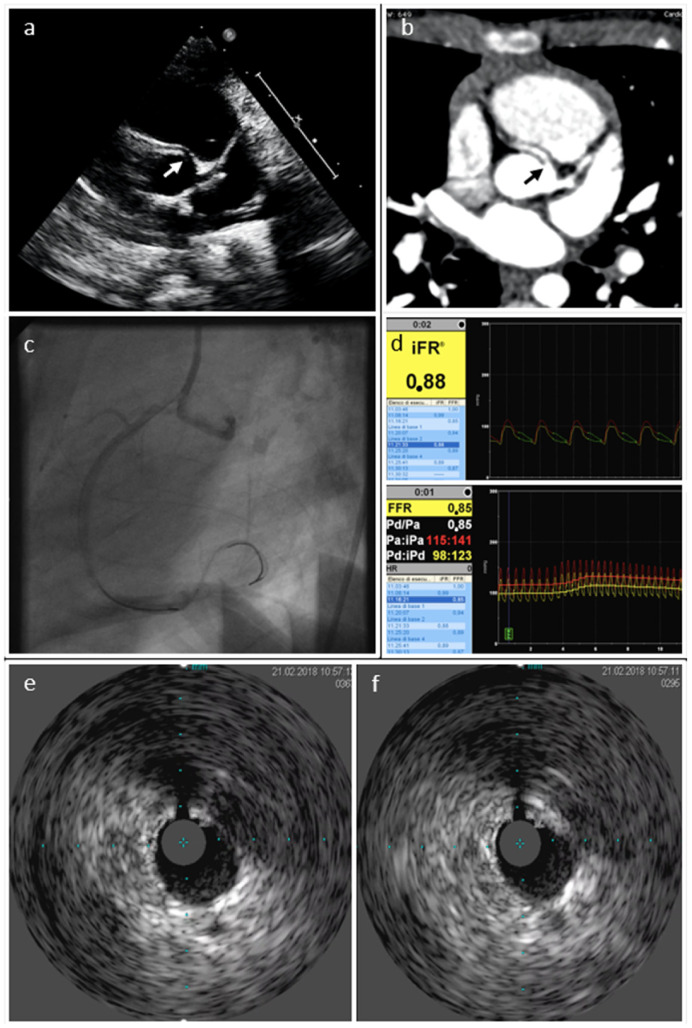
A 7-year-old patient with chest pain. Ultrasound examination showed a right coronary artery from the left sinus (**a**), confirmed by computed tomography ((**b**) arrow); coronary angiography (**c**) showed an iFR in the grey zone (**d**); intravascular ultrasound showed a ring of tissue around the ostium of the right coronary artery with systolic contraction, attributable to intramural course (**e**,**f**).

iFR is calculated as the ratio of blood pressure distal and proximal to a coronary artery stenosis during the diastolic wave-free period, which starts at 25% of the diastolic phase and ends 5 ms before systole, when the microvascular resistance result is steadily low; it might be more indicative of the effective myocardial perfusion. In adults with plaque-related ischemia the test is positive for values < 0.86, and results between 0.93 and 0.86 are considered in the “grey zone” [170]. However, frequently a single cutoff of 0.89 is used to define hemodynamic significance [172,173,174] (Figure 24).

During the procedure, if a woven-like coronary artery is detected, intravascular imaging is essential for accurate differential diagnosis between congenital anomalies, spontaneous coronary artery dissection, or thrombus recanalization [175] ] (Table 4). Moreover, automated IVUS image processing and quantification of CAAs is reported to enhance the evaluation of geometrical changes in coronary vessels in CAA patients and enable efficient clinical analysis in a streamlined workflow [176,177]. Furthermore, IVUS can provide good procedural guidance during complex percutaneous coronary intervention (PCI) procedures in patients with CAAs, such as confirming optimal stent deployment [178].

## 7. Surgical Treatment and Management

CAA management and its potential surgical treatment are strictly related to its anatomical location, hemodynamic implications and associated patient symptoms [93,179].

Regarding AAORCA, asymptomatic patients without high-risk features may undergo periodic surveillance; however, functional testing is recommended as evidence of ischemia in territories subtended by the anomalous coronary and/or high-risk anatomical findings should prompt intervention [94]. In fact, symptomatic individuals or those with demonstrable ischemia or malignant anatomical features are often considered for surgical correction such as unroofing of the intramural segment, which is the most commonly performed surgical procedure, with the aim to create a new, physiologically open ostium in the appropriate sinus, opening the shared wall between the aorta and the intramural coronary tunnel [180,181]. However, when the coronary artery origin is too close to the commissure for unroofing to be performed without risk or is too distant from the ideal site, coronary reimplantation seems to be the best option with an anatomical correction performed reattaching the excised anomalous coronary origin to the appropriate aortic sinus [180,182]. When the ostial morphology remains restrictive, ostioplasty can be associated with enlargement of a slit-like or narrowed coronary ostium [180]. Rarely, in case of an interarterial course, when unroofing and or reimplantation are not feasible or deemed high risk, pulmonary artery translocation could be performed to increase the space between the main pulmonary artery and the aorta reducing the extrinsic pressure and possible compression. Coronary artery bypass grafting can be suggested when reimplantation is anatomically unfeasible, when the proximal segment is long and intramural, in case of intraoperative complications during reconstructive procedures, or in adult patients with coexisting obstructive coronary artery disease [180,183].

Regarding AAOLCA, current guidelines generally recommend surgical repair for symptomatic and asymptomatic patients with high-risk anatomical features; obviously, the strongest recommendations regard patients with coronary ischemia attributable to the anomalous coronary artery. However, in asymptomatic patients without evidence of myocardial ischemia, surgery may be considered too [94]. As for AAORCA, surgical options include unroofing, ostioplasty, reimplantation and bypass grafting [180,184,185,186]. Moreover, transeptal course can be treated with coronary artery bypass graft, mobilization of the pulmonary root and incision of the overlying muscle bridge with translocation of the right pulmonary artery, or a transconal approach with transection or unroofing of the LMCA or LAD septal course, followed by tract repair of the posterior wall of the right ventricle outflow with an autologous pericardial patch [85].

Regarding anomalous origin of the LCx from the right sinus or right coronary artery, routine clinical follow-up is sufficient, in master athletes too, with intervention reserved for unusual presentations or coexistent obstructive disease [72,187].

SCA risk stratification is almost entirely based on the trajectory, ostial characteristics and patient’s symptoms. Malignant courses are usually candidates for surgical repair, whereas benign subtypes usually require only observation. However, the patient’s age, comorbidities, and invasive hemodynamic testing are taken into account to evaluate surgical eligibility in the context of a multidisciplinary approach and risk–benefit discussion with the patient [73,188].

Regarding ALCAPA, surgical reimplantation of the left coronary artery into the aorta is the recommended therapy. Without surgical correction, mortality exceeds 80% in infancy; moreover, concomitant moderate or severe mitral valve regurgitation should undergo mitral valve operation at the same time [189,190,191]. Surgical reimplantation of the anomalous right coronary artery to the ascending aorta is the most common method of repair in ARCAPA patients, particularly if symptomatic or with proven ischemia, with excellent long-term outcomes [192,193].

Regarding high-takeoff coronary arteries, most cases require no intervention; however, surgical or catheter-based strategies may be needed in the setting of associated myocardial ischemia, significant ostial narrowing or procedural complications [17,75,77].

Separate origins of LAD and LCx do not require specific interventions beyond standard cardiovascular care, but special maneuvers during guide catheter management for percutaneous intervention are needed [19,108,194].

First-line treatment of myocardial bridging is medical, based on beta-blockers, with calcium-channel blockers and nitrates usually considered as alternatives [195,196]; surgical options (myotomy or coronary artery bypass graft) are reserved for refractory cases, and coronary artery bypass graft seems to be preferred over myotomy when the myocardial bridge is longer than 25 mm, deeper than 5 mm or when the spanned coronary segment fails to completely decompress in diastole [197]. Regarding intracavitary coronary arteries, there is no evidence that medical treatment is beneficial given the absence of mechanical compression by the heart muscle. Intracavitary coronary arteries can be moved to a more aerial position or an anastomosis can be performed inside the cavity or with a more distal epicardial vessel segment [35].

No specific treatment is generally required in coronary crossing and coronary artery duplication, but recognizing the anomalous anatomy is crucial in the decision-making process of revascularization [30,198,199,200].

Trans-catheter coiling can be performed to treat coronary-to-pulmonary artery fistulas [201]. Symptomatic coronary fistulas can be surgically treated by ligation of the aberrant vascular branch at the drainage site [43].

Three to six months after surgery, CCTA or CMR is performed to confirm patency. Stress testing (exercise electrocardiography, echocardiography, or perfusion imaging) is suggested to confirm the absence of ischemia under physiologic stress, particularly in patients with silent ischemia or myocardial fibrosis on preoperative imaging. Fibrosis can be evaluated by cardiac MRI late gadolinium enhancement, and when detected prolonged rhythm monitoring may be warranted to assess arrhythmia risk [180].

## 8. Discussion

CAAs can be benign or malignant, with the latter being associated with high-risk features such as ALCAPA, coronary origin from an opposite aortic sinus or non-coronary sinus with an interarterial or intramural course, slit-like origin or acute angle of takeoff, myocardial bridging with a bridge depth higher than 5 mm or an associated milking effect and myocardial hypoperfusion, or coronary artery fistula with significant vascular shunt [1,43]. In fact, CAAs are reported as a not-infrequent cause of sudden cardiac death in young athletes [1,202]. Moreover, cardiac and extracardiac anomalies can be associated with CAAs and should be excluded before surgery or invasive procedures [15,203].

Transthoracic echocardiography is the first-line imaging modality, as it can diagnose an abnormal coronary origin and can raise suspicion of the presence of other anatomic features that can influence management decisions such as intramural course and interarterial course [186]. When transthoracic echocardiography images are suboptimal and in patients with symptoms suggestive of ischemia, other imaging modalities can be used to confirm the diagnosis, such as transesophageal echocardiography, computed tomography and magnetic resonance imaging [204].

Retroaortic, transseptal/subpulmonic, and prepulmonic course identification remains crucial, especially in preoperative settings or in patients undergoing invasive procedures such as coronary artery bypass grafting or aortic valve replacement, to prevent surgical errors or iatrogenic injury [94,205]. Moreover, transseptal/subpulmonic was associated with a higher risk of symptoms and sudden cardiac death; therefore, in the case of evidence of ischemia surgical repair would be indicated [85].

In myocardial bridging, it is particularly important to document the extent of systolic compression and its potential hemodynamic impact—especially in symptomatic patients or those with inducible ischemia [116,197].

CCTA increases sensitivity for clinically relevant features (interarterial versus benign courses, intramural length, intracavitary course, and compression between great vessels) and improves preoperative planning by providing surgeons and interventionists with accurate 3D maps that inform decisions about unroofing, reimplantation, or conservative management. It also supports noninvasive follow-up: high-resolution CCTA (and, in time, PCCT) can be used to monitor postsurgical anatomy, detect recurrent narrowing, and evaluate adjacent aortic or pulmonary root changes without subjecting patients to repeated invasive angiography [206]. In pediatric patients with CAAs, CCTA plays a particularly strategic role as it provides a comprehensive, noninvasive assessment of coronary anatomy in a single acquisition, avoiding the risks and limitations of invasive angiography in small-sized individuals. In children, the main challenges include very small coronary diameters, high heart rates, and heightened sensitivity to the cumulative risks of ionizing radiation. The use of dual-source CT enables high-quality imaging even at heart rates >100 bpm without the need for deep sedation or beta-blockers, while iterative reconstruction techniques maintain diagnostic image quality at radiation doses often below 1 mSv in optimized protocols. The emergence of PCCT offers further potential advantages in this setting: improved spatial resolution and reduced image noise can enhance the depiction of small coronary lumina and detailed characterization of ostial morphology and vessel course—critical for distinguishing high-risk anomalies from benign variants. Moreover, in children who have undergone surgical or interventional correction of a coronary anomaly, CCTA is well suited for periodic follow-up, minimizing radiation exposure and avoiding vascular access while accurately monitoring vessel patency, restenosis, or geometric changes in the coronary arteries in relation to somatic growth [207,208]. In competitive athletes, CT helps to differentiate benign variants from potentially malignant configurations, guiding eligibility and return-to-play decisions [209]. Limitations remain because temporal resolution of CT still cannot match the instantaneous frame rates of catheter angiography and CCTA can be challenged by arrhythmias, extremely high heart rates, or severe coronary calcification; access to PCCT is currently limited and vendor-specific differences in IR and spectral implementations mean that protocols must be tailored and validated locally. Nevertheless, the net effect of DSCT, advanced IR and emerging PCCT is a meaningful stepwise increase in diagnostic performance: fewer non-diagnostic scans, better definition of high-risk anatomical features, lower radiation burden and improved confidence in both detection and characterization of coronary anomalies, which ultimately improves triage and management. Recent advances expand CCTA beyond morphology into functional assessment. CT-derived FFR and computational fluid dynamics permit noninvasive evaluation of flow restriction [210,211]. CT perfusion complements these findings by identifying dynamic ischemia [212,213,214]. Integration of anatomical and functional data—such as CT-FFR, perfusion imaging, and wall shear stress mapping—enhances the ability of CCTA to serve as a single comprehensive tool for both diagnosis and risk assessment [215].

While CCTA is often preferred for detailed anatomical definition, CMR provides crucial information regarding the functional and physiological consequences of coronary artery anomalies. Through phase-contrast sequences, CMR enables quantitative assessment of blood flow across coronary fistulas or shunts, allowing estimation of the associated hemodynamic burden. At the same time, it permits accurate evaluation of ventricular volumes, wall thickness, and systolic function, which is essential for clinical decision-making and for monitoring disease progression [90,152,216].

Furthermore, CMR allows assessment of myocardial perfusion. Stress perfusion imaging can detect inducible ischemia related to coronary steal phenomena, particularly in the presence of large or high-flow fistulas, even in the absence of obstructive coronary artery disease. In addition, late gadolinium enhancement sequences enable the identification of myocardial fibrosis or scarring, providing evidence of chronic myocardial injury and potential long-term sequelae [152,217].

Another important role of CMR is in patient follow-up. The technique allows safe, repeatable evaluation of coronary dilation, shunt flow, and ventricular remodeling after conservative management or interventional closure, without exposure to ionizing radiation. This feature is especially advantageous in pediatric patients and in individuals requiring serial imaging over time [217,218]. Obviously, when necessary, imaging follow-up can be performed with CTCA, such as when an early post-surgical complication needs to be excluded [219].

Regarding clinical applications of nuclear medicine, in coronary arteries with interarterial/intramural anomalies, functional testing that could demonstrate ischemia is central to therapeutic decisions; in fact, exercise stress testing with SPECT or pharmacologic stress PET with MBF quantification may identify inducible ischemia or reduced MFR, supporting surgical correction in symptomatic patients or selected asymptomatic patients with objective ischemia [94,220]. In ALCAPA, which typically presents in infancy with left ventricular dysfunction and ischemia, in addition to anatomical imaging, nuclear perfusion and FDG viability imaging can identify viable myocardium and delineate infarcted areas, aiding the timing of surgical reimplantation and predicting postoperative recovery [221]. Large coronary fistulas may produce the steal phenomenon, and myocardial perfusion imaging can quantify its hemodynamic significance and guide closure timing [222]. Single coronary artery anomalies require functional assessment to confirm adequate perfusion of dependent myocardial territories [188].

Moreover, in some cases, fusion imaging combining CT with CMR or 3D echocardiography could facilitate disease assessment and preoperative planning [223,224].

Fractional flow reserve and intravascular ultrasound-guided management in adult patients is suggested [225].

Future developments combining photon-counting CT with AI-driven hemodynamic modeling are expected to enable personalized evaluation of congenital coronary anomalies and refine surgical planning. In the NARCO trial, patients with an anomalous aortic origin of the coronary artery will undergo CCTA to define characteristics of anatomical high-risk features (using CCTA) to rule out noninvasively hemodynamically relevant anomalous vessels in these patients and to help in optimizing patient selection for revascularization and, therefore, prevent unnecessary downstream testing and/or costly interventions [226]. Future studies are needed to better evaluate the potential for fluid–structure interaction modeling to guide surgical management noninvasively [227]. Further longitudinal studies about exercise stress CMR are warranted with larger sample sizes in multiple institutions to confirm the feasibility of this technique to assess for myocardial ischemia with exercise in this at-risk population. Future studies need to better define genetic predisposition and associated syndromes, in order to try to enhance coronary artery anomalies and anatomical variant detection in specific populations. Moreover, confocal Raman spectroscopy imaging as a high-resolution tool for assessing cardiac pathology should be better evaluated in future studies to confirm its potential to revolutionize the early diagnosis and real-time monitoring of myocardial remodeling [228]. Furthermore, new strategies of construction of myocardial ischemia and infarction models for preclinical research could be evaluated in future studies [229]. Finally, serum metabolic fingerprints should be extensively tested in clinical practice to evaluate the potential to reduce time spent in the emergency department and improve treatment for myocardial ischemia and infarction [230].

## 9. Conclusions

Some coronary anomalies are life-threatening and their diagnosis allows correct treatment to reduce possible anomaly-related deaths. Instead, a lot of coronary anomalies are considered benign and without associated symptoms; however, a precise anatomical and functional assessment is essential to ensure safe and effective interventional and surgical planning. Therefore, coronary artery imaging is very important and should be suggested before invasive procedures for proper planning and to significantly reduce treatment-related arterial complications. Ultrasound is frequently the first imaging that could raise suspicion of a coronary anomaly, particularly if the origin is anomalous. Computed tomography remains the reference standard for anatomical characterization, and frequently an anomalous coronary course is incidentally detected during a coronary examination. Usually, cardiac magnetic resonance imaging offers a complementary and comprehensive perspective by integrating assessment of flow dynamics, myocardial perfusion, tissue characterization, and ventricular function, thereby helping to define the clinical significance of coronary artery anomalies and to guide management strategies. However, it should be noted that nowadays recent computed tomography technical advancements allow us to obtain a huge amount of this functional and perfusion information, but with a slightly higher radiation and contrast media dose. SPECT and PET can sometimes provide information on CAA functional implications such as regional myocardial perfusion, ischemia, viability, and physiological flow reserve. However, an expert clinical evaluation is necessary to evaluate symptoms and imaging findings together and decide when to proceed further with invasive imaging and/or surgical operation to reduce patient discomfort or the risk related to some of those life-threatening diseases. Moreover, cardiac and extracardiac anomalies can be associated with CAA and should be excluded before surgery or invasive procedures.

## Figures and Tables

**Table 1:** Coronary artery anomaly epidemiology.

Anomaly or Variant	Prevalence (%)	Key Features
Right-sided anomalies of origin	0.5	Abnormal origin; sometimes clinically relevant, particularly with interarterial course
Left-sided anomalies of origin	0.1	Abnormal origin with possible high-risk features depending on ostium characteristics and course
Single coronary artery	0.05	Only one artery; sometimes malignant course
Left coronary artery from the pulmonary artery	0.01	Flow from high- to low-pressure systems with chronic myocardial ischemia and possible death
Right coronary artery from the pulmonary artery	0.002	Flow from high- to low-pressure systems with chronic myocardial ischemia
Myocardial bridge	5	Myocardial hypoperfusion possible when the bridge is longer than 2.5 mm and its depth is at least 2 mm
Coronary fistulas	0.4	Coronary artery drains into an atypical cardiac or extracardiac structure, sometimes symptomatic

**Table 2:** Characteristics of common PET myocardial perfusion tracers.

Tracer	Physical Half-Life	Production Method	Mechanism of Uptake	Main Advantages	Main Limitations
[13N]Ammonia	9.96 min	Cyclotron	Passive diffusion followed by metabolic trapping in myocardial cells	High extraction fraction, excellent image quality, validated quantification	Requires on-site cyclotron, short half-life limits availability
[82Rb]Cloruro	1.26 min	Generator (82Sr/82Rb)	Potassium analog, active uptake via Na^+^/^++^ ATPase pump	Readily available, generator-produced, quick protocols	High positron range (lower resolution), limited quantification accuracy
[15O]Water	2.03 min	Cyclotron	Freely diffusible, reflects true perfusion	Gold standard for MBF quantification, linear relation with flow	Requires cyclotron on-site, no myocardial retention (quantifying myocardial perfusion requires specialized kinetic software)
[18F]Flurpiridaz	109.7 min	Cyclotron	Binds to mitochondrial complex I	Excellent image quality, long half-life allows regional distribution, high extraction	It is still undergoing a Phase III clinical trial in the European Union and is not yet approved for medical use

**Table 3:** Comparison of SPECT and PET.

Parameter	SPECT *	PET *
Primary Tracers	99mTc-sestamibi, 99mTc-tetrofosmin, 201Tl	[13N]Ammonia, [82Rb]Cloruro, [15O]Water, [18F]Flurpiridaz
Type of Measurement	Relative perfusion	Absolute perfusion (MBF *, MFR *)
Spatial Resolution	8–12 mm	4–6 mm
Attenuation Correction	Optional (SPECT/CT hybrid)	Standard (PET/CT, PET/MRI)
Quantification Accuracy	Semi-quantitative	Fully quantitative
Acquisition Time	15–20 min	10–15 min
Effective Dose	7–10 mSv	2–4 mSv

* SPECT, single photon emission computed tomography; PET, positron emission tomography; MBF, myocardial blood flow; MFR, myocardial flow reserve.

**Table 4:** Coronary artery anomalies imaging modalities.

Anomaly or Variant	Ultrasound	Computed Tomography	Magnetic Resonance Imaging	Nuclear Medicine	Coronary Angiography
Right-sided anomalies of origin	Can suspect its diagnosis and a malignant course	Diagnostic; morphological and potential functional information; good assessment of coronary course	Diagnostic; morphological and functional information; usually, worse course evaluability than computed tomography	Non-diagnostic; can detect stress-induced ischemia	Diagnostic; invasive functional data; ostial narrowing and intramural segment detection better evaluable with intravascular ultrasound
Left-sided anomalies of origin	Can suspect its diagnosis and a malignant course	Diagnostic; morphological and potential functional information; good assessment of coronary course	Diagnostic; morphological and functional information; usually, worse course evaluability than computed tomography	Non-diagnostic; can detect stress-induced ischemia	Diagnostic; invasive functional data; ostial narrowing and intramural segment detection better evaluable with intravascular ultrasound
Single coronary artery	Absence of a coronary artery	Diagnostic; morphological and potential functional information; good assessment of coronary course	Diagnostic; morphological and functional information; usually, worse course evaluability than computed tomography	Non-diagnostic; can detect stress-induced ischemia	Diagnostic; invasive functional data; ostial narrowing and intramural segment detection better evaluable with intravascular ultrasound
Left coronary artery from the pulmonary artery	Absence of a normal left coronary artery origin	Diagnostic; morphological and potential functional information; good assessment of coronary course	Diagnostic; morphological and functional information; usually, worse course evaluability than computed tomography	Non-diagnostic; can detect stress-induced ischemia	Diagnostic; invasive functional data
Right coronary artery from the pulmonary artery	Absence of a normal right coronary artery origin	Diagnostic; morphological and potential functional information; good assessment of coronary course	Diagnostic; morphological and functional information; usually, worse course evaluability than computed tomography	Non-diagnostic; can detect stress-induced ischemia	Diagnostic; invasive functional data
Myocardial bridge	Usually non-diagnostic	Diagnostic; morphological and potential functional information	Usually non-diagnostic; functional information	Non-diagnostic; can detect stress-induced ischemia	Diagnostic, but lower sensitivity than computed tomography; invasive functional data
Coronary fistulas	Usually non-diagnostic	Diagnostic; morphological and potential functional information	Usually non-diagnostic; functional information	Non-diagnostic; can detect stress-induced ischemia	Diagnostic; invasive functional data

## Data Availability

No new data were created or analyzed in this study. Data sharing is not applicable to this article.

## Abbreviations

The following abbreviations are used in this manuscript:

CAA	Coronary artery anomaly
AAORCA	Anomalous aortic origin of the right coronary artery
RCA	Right coronary artery
CCTA	Coronary computed tomography angiography
CMR	Cardiac magnetic resonance
AAOLCA	Anomalous aortic origin of the left coronary artery
LMCA	Left main coronary artery
LCx	Left circumflex artery
SCA	Single coronary artery
ALCAPA	Anomalous origin of the left coronary artery from the pulmonary artery
ARCAPA	Anomalous origin of the right coronary artery from the pulmonary artery
ACXPA	Anomalous circumflex coronary artery connected to the pulmonary artery
ALADPA	Anomalous left anterior descending coronary artery connected to the pulmonary artery
ASCAPA	Anomalous single coronary artery connected to the pulmonary artery
LAD	Left anterior descending
MB	Myocardial bridging
CT-MPI	CT-myocardial perfusion imaging
SPECT	Single photon emission computed tomography
iFR	Instantaneous wave-free ratio
SCD	Sudden cardiac death
FFR	Fractional flow reserve
PSAX	Parasternal short axis
PLAX	Parasternal long axis
RAC	Retro-aortic sign
DSCT	Dual-source CT
IR	Iterative reconstruction
PCCT	Photon-counting detector CT
bSSFP	Balanced steady-state free precession
iNAV	Image-based navigator
dNAV	Diaphragmatic navigation
sNAV	Self-navigation
PET	Positron emission tomography
MPI	Myocardial perfusion imaging
MBF	Myocardial blood flow
MFR	Myocardial flow reserve
IVUS	Intravascular ultrasound
PCI	Percutaneous coronary intervention
